# Motivating Transparent Communications about Bias in Healthcare Technology Development

**DOI:** 10.1525/collabra.136456

**Published:** 2025-05-06

**Authors:** Anna Tovmasyan, Alice Liefgreen, Sandra Wachter, Brent Mittelstadt, Netta Weinstein

**Affiliations:** 1 School of Psychology and Clinical Language Sciences, University of Reading; 2 Language and Cognition, University College London; 3 Oxford Internet Institute, University of Oxford

**Keywords:** healthcare AI, transparency, inclusion, motivation, values

## Abstract

As healthcare artificial intelligence (AI) systems advance, their capacity for bias (e.g., as a function of patient protected characteristics) increases as well and these limitations are often left undisclosed by developers. Here, the question arises - do supportive motivational messaging designed to increase buy-in inspire healthcare AI developers to transparently communicate about bias in their technology? Computer science students (Study 1: *N*=271; Study 2: *N*=209) were randomly assigned to receive a brief communication framed in either an autonomy-supportive (choice promoting) or controlling (judging and pressuring) way, emphasizing either personal benefits (gaining profit) of transparency or legal implications of non-transparency. Results showed that while communication type was not associated with behavioral intention to engage in an educational course on transparent communication about bias, both internal (self-directed) and external motivations were associated with greater intention to take a course to build transparency-congruent technology skills, as well as greater ethical voice - intention to speak up in the service of positive transparency-consistent cultural change, and lower antagonism – i.e., a lower critical perspective regarding the need for transparency. Findings suggest that universities and workplaces should provide students and developers with a broadly supportive motivational climate, rather than providing a singular brief training.

Commercial organizations that develop healthcare artificial intelligence (AI) may hold values such as transparency and fairness, but the reality of product development often stands in the way of behaving in line with those values, potentially resulting in producing biased algorithms. Bias can arise from various factors, such as the prioritization of efficiency and profitability over fairness and transparency, or the reliance on biased training data. For example, biased AI in healthcare has led to disparities in resource allocation and diagnostic accuracy across different demographic groups (e.g., Obermeyer et al., 2019). Addressing these issues requires not only technical fixes, but also a cultural and motivational shift within organizations.

One potential solution lies in educational initiatives designed to raise awareness of AI bias and inspire value-consistent behavior among developers. However, achieving widespread enrollment and meaningful engagement with such courses is challenging, particularly in an environment where external pressures and incentives dominate decision-making (Rakova et al., 2021). Motivational principles that inspire ‘buy-in’ or are autonomy-supportive and inspire greater personally-based (i.e., identified) motivation rather than relying on punishment and pressure (i.e., external motivation) have been suggested as an approach to inspiring investment in value-consistent behaviors (Liefgreen et al., 2023).

The current experiments explored this possibility using brief videotaped communications based on other studies of behavioral change that apply those motivational principles (Legate et al., 2022) and examined whether motivational framing generalizes both when communicating laws related to fairness expression or personal benefit of value expression (two important ways to communicate punishment or reward for behavior; Liefgreen et al., 2023). We specifically examined whether brief communications could shape motivation to follow one’s values and value-consistent behavioral intention, or whether instead, individual differences in motivations might better relate to behavioral intention.

## The Importance of Values in Healthcare AI

AI systems hold the potential to make decision healthcare processes more efficient and accurate (Chung et al., 2020). They could also help to reduce or even eliminate conscious or unconscious human bias, which often hinders the process of fair decision-making with substantial implications for patient care (Johnson, 2020; Stone & Moskowitz, 2011). However, while using AI could be a promising method of reducing such bias, AI itself is not entirely bias-free (see Challen et al., 2019). For example, Obermeyer et al. (2019) found that a healthcare algorithm disproportionately allocated fewer resources to Black patients compared to White patients due to biases embedded in the training data. Similarly, Buolamwini and Gebru (2018) demonstrated that facial recognition systems exhibit higher error rates for darker-skinned individuals and women, raising questions about the representativeness of the datasets used to train these models.

Biases in AI systems could arise from a range of sources including the algorithm, input features chosen, having small or incomplete training datasets, or hidden correlations within these datasets (Mehrabi & Moayedi, 2021). When fed into real-world systems, such as medical fields (Hamet & Tremblay, 2017) as well as courtrooms (Campbell, 2020) or childhood welfare systems (Hunt, 2020), the outcomes of these algorithms can have detrimental consequences unless they are designed correctly and fairly (Mehrabi & Moayedi, 2021). While larger organizations may have structures in place for ethical oversight, such as employing ethicists, these mechanisms are not always sufficient to address the complex ethical challenges inherent in AI development. For example, Ryan et al. (2021) highlight that many organizations, including those with significant resources, currently lack sufficient ethical training for their teams. Therefore, while larger organizations may be better positioned to implement regulatory requirements due to their resources, smaller organizations, especially those with limited capacity, likely find it more challenging to meet ethical standards. This disparity in resources and oversight capacity underscores the systemic challenges that exist in ensuring ethical AI development across the industry. These challenges highlight the need for broader solutions, including accessible training and oversight mechanisms for organizations of all sizes.

Additionally, developers may not intentionally avoid addressing bias but could be unaware of its presence or lack the tools to detect it effectively (Holstein et al., 2019). Further, although developers are often asked to extend their best effort to reduce bias introduced by the algorithms, it may not always be easily feasible or attractive to do so; developing fair and unbiased technology requires heavy financial investment and comes with costs to product development timelines and reputation (Buckley et al., 2021). As such, taking healthcare AI as an example, data that are being imputed often predominantly reflect information about White patients, yielding more reliable technology for them but not necessarily for patients of other ethnicities (Norori et al., 2021). This could happen because historical healthcare data often reflects existing societal inequalities, such as disparities in access to healthcare or underrepresentation of certain demographic groups (Kenworthy et al., 2020). As a result, AI models trained on such data may perpetuate these biases, leading to inaccurate or unequal treatment recommendations (Rajkomar et al., 2018). This highlights the importance of educational initiatives that raise awareness of AI bias and provide developers with the motivation and tools to mitigate it.

Decision-making transparency in healthcare AI is fundamental for enhancing public trust and ensuring patient safety (Ross & Spates, 2020). Transparency enables healthcare professionals, regulators, and patients to understand the underlying mechanisms, assess biases, and identify potential errors or limitations (Obermeyer et al., 2019; Rajkomar et al., 2018). However, healthcare AI developers frequently lack transparency when it comes to disclosing the limitations of their technology, posing significant concerns for patient safety and informed decision-making. Many commercial AI algorithms for medical imaging lack transparency in their documentation, failing to provide comprehensive information about the data used, algorithmic details, or potential limitations (Char et al., 2018). This can lead to overreliance on AI systems, with healthcare professionals and patients being unaware of their limitations, potentially resulting in misdiagnoses, inappropriate treatments, or compromised patient safety. Yet, developers may not always be motivated to be transparent, and identifying incentives that could drive developers to be transparent is essential. In short, healthcare technology industry faces a great challenge, how to motivate or inspire developers to engage in more value-consistent behaviors that give expression to values such as transparency and inclusive practice; using brief communications derived from public health practices this may be achievable *en masse* over social media, and through organization-level statements and educational materials.

### Motivating Engagement with Anti-Bias Education

Addressing bias in AI systems is a critical challenge that requires multifaceted solutions, including robust regulatory frameworks, systematic auditing processes, and developer engagement. While regulations and audits serve as essential mechanisms to ensure compliance and accountability, it is equally important to instil awareness among students who are training to become developers about the biases that can infiltrate AI systems. By beginning this education early, students can develop the skills and mindset needed to prioritize fairness and transparency throughout their careers in AI development.

Evidence from related fields, such as diversity training and ethics education, suggests that targeted training can improve awareness, attitudes, and practices related to bias (Bezrukova et al., 2016; Stolt et al., 2018). However, barriers to participation in such programs persist. These challenges are especially pronounced for students, who may not yet see the direct connection between issues of bias and their future roles as developers. Addressing these motivational barriers is vital for developing engagement with educational programs that promote transparency and fairness in AI development.

Evidence from studied following the framework of self-determination theory (Ryan & Deci, 2000) demonstrates the importance of aligning interventions with personal values to sustain motivation and meaningful behavior change (see Teixeira et al., 2020). Brief communications that emphasize values such as transparency and inclusion have shown promise in influencing attitudes and behaviors across diverse contexts, including within police-force (Al-Khouja et al., 2020; Weinstein et al., 2023) and public health (Altendorf et al., 2019) settings. These findings suggest that concise, well-crafted messages could serve as a scalable and cost-effective method to encourage students to engage with educational content on bias and fairness.

By addressing student motivations directly, brief motivational communications provide an opportunity to bridge the gap between awareness and action. These messages can serve as a first step in cultivating engagement with comprehensive educational programs aimed at developing the technical and ethical competencies needed to identify, mitigate, and communicate potential sources of bias. The present study builds on this foundation by exploring how to design such communications effectively, and through evaluating their impact on students’ willingness to participate in anti-bias training programs, setting the stage for long-term improvements in transparency and fairness in AI development.

### Motivating Buy-In for Healthcare AI Development

We rely on SDT (Ryan & Deci, 2000) as a framework for exploring how brief motivational communications can inspire buy-in - willingness to voluntarily adopt transparency values - when communicating about bias in healthcare technology. This study distinguishes between two forms of communication: autonomy-supportive (buy-In) and controlling (mandates). Autonomy-supportive communications motivate through making choiceful behavior salient, align behavior with values, and provide compelling reasons for actions that help get recipients on board with those actions (deCharms, 1968; Ryan & Deci, 2000). In contrast, research has shown that controlling communications, those that motivate through creating feelings of shame, guilt, and pressure, might decrease engagement and ultimately yield ineffective or even counterproductive results (Legault et al., 2011). Autonomy-supportive, as opposed to controlling communications, have been found to be associated with greater enhanced performance and persistence, more in-depth information processing, and greater well-being (Cooper et al. 1995; Vansteenkiste et al. 2005). Research further suggests that such supportive communications foster a positive environment that encourages individuals to openly discuss challenges and drawbacks and increases their willingness to disclose concerns or limitations (Deci et al., 1989; Lee & Kim, 2021). Given this work, autonomy-supportive communications may be especially helpful when making a difficult but value-laden decision to invest in transparent communications. Research comparing these two forms of communications highlights that autonomy-supportive are more effective at attaining desired behavioral outcomes as compared to controlling communications (Weinstein et al., 2023). However, current research tends to focus on sports and health behavior (e.g., Celio et al., 2017; Legate & Weinstein, 2022; Ntoumanis et al., 2017), and little work has been conducted in relation to value-laden behavior, such as in the case of mitigating harms of bias and pursuing transparency (World Health Organization, 2021).

### Internal and External Motivations Differentially Relate to Action

The current study examines how the two forms of brief communication - autonomy-supportive and controlling - may impact motivations to engage in an educational course designed to educate technology students to be transparent about potential bias in technology. SDT (Deci & Ryan, 1985) delineates two types of motivation that energize action: internal and external, and furthermore research from SDT has shown that autonomy-supportive versus controlling motivations promote self-directed internal *behavioral regulation* (i.e., motivation) that come from within individuals and reflect their interests and values (e.g., Grolnick et al. 1991; Gagné et al. 2003),

Internal motivation, which refers to a self-driven desire to act because one sees the value and importance of the activity, is considered to be the best source of sustained and commitment engagement in the motivated domain of (Deci & Ryan, 1989). It follows that if technology developers consider transparency to be a personal value that is important to who they are (i.e., their identity), they would be more likely to act in line with this value. Given that internal motivation has also been associated with increased task engagement even when possible costs are at stake (Lepper et al., 1973), developers may be more willing to transparently report the drawbacks of their technology if they are internally motivated to do so.

Contrary to internal motivation, external motivation refers to motivation emerging either from external forces, including costs or benefits that drive individuals to engage in an activity, or internalized self-imposed costs or benefits such as shame, guilt, or pride (Deci & Ryan, 1989). Mandates, such as regulatory requirements or industry standards, can also act as powerful external motivators, as they impose clear consequences for non-compliance. Indeed, as commercial entities, healthcare AI developers are often driven by financial gains and the potential for market success. While communicating the capabilities of their technology and being silent about the drawbacks could seem as a logical and attractive strategy to increase profit, developers who prioritize transparency are more likely to gain the trust of healthcare providers, regulators, and patients (Matheny et al., 2019), leading to increased adoption and market success (Gerke et al., 2020). Thus, emphasizing the importance of transparency in the pursuit of profit can act as a strong external motivator for healthcare AI developers, ultimately benefiting both their financial goals and the overall advancement of responsible AI in healthcare.

### Profit and Legal Motives for Action

Alongside motivational framing, laws may also be put in place to create clear negative contingencies on behavior not aligned with values; these have already been developed for fairness and transparency values in the AI healthcare realm. Specifically, according to anti-discrimination laws and principles of fairness, equal treatment across groups is not enough - this treatment should also produce equal outcomes (Equality Act, 2010). In the context of healthcare, this can apply to medical diagnostic tools or treatments that may unintentionally exhibit racial or gender disparities. The European Union’s General Data Protection Regulation (GDPR) highlights the need for transparency and justification when automated decision-making, including medical diagnostic tools, disproportionately affects certain groups. These legal frameworks emphasize the importance of addressing and justifying disparities in healthcare practices to ensure fairness and equal treatment for all individuals. Communicating about legal implications may be more effective when the communication is autonomy-supportive, helping developers to buy-in to the law itself (Liefgreen et al., 2023). But does the same motivational principle apply when encouraging developers to think about the personal benefits (in terms of rewards) of value-laden behaviors, which may inspire buy-in themselves? To explore these dynamics, this study compares the effectiveness of motivational framing based on profit (self-interest) versus law (legal imperatives) in encouraging intention to engage in an educational course about transparency in AI systems.

### The Current Research

The current experiments examined motivational framing for communication (autonomy-supportive versus controlling) and external motives embedded within communications (personal profit versus law) to promote various types of motivation and behavioural intention to engage in further education about transparency in communicating about bias in technology. While organizations use brief messages to obtain desired employee’s behavior and such messages allow for widespread communication across large and dispersed stakeholders, the efficacy of such interventions remains unclear. The current studies were conducted with students of technology rather than professionals in the technology development space to minimize the existing previous knowledge on the topic in order to avoid the confounding factors (see Legate et al., 2022).

We designed the experiments to address the following question: does the framing of communications that encourage students to transparently communicate the bias in technology affect intention to behave in line with transparency values? We pre-registered the following hypotheses:

H1: The Buy-In (i.e., autonomy-supportive) conditions will predict more positive behavioural intention when compared to the Mandates (i.e., controlling) conditions.

H2: Those in profit condition (i.e., where self-interest is highlighted) will exhibit more positive behavioural intention than those in law condition (i.e., where potential losses are highlighted).

H3: Buy-in would magnify beneficial effects of law on positive behavioral intention and beneficial effects of self-interest on positive behavioral intention.

## Methods and Results Across Studies

### Transparency and Openness

We report how we determined our sample size, all data exclusions, manipulations, and measures in the study. The design, methodology, hypotheses, and analyses of this study were pre-registered on the Open Science Framework on February 20th, 2023. All materials, data, and analyses scripts are available on the Open Science Framework: https://osf.io/5uq27/?view_only=1a92cc38b2974dc2a28485610711a81d.

### Participants and Procedure

All participants were treated in accordance with American Psychological Association ethical guidelines for research (Sales & Folkman, 2000) and the World Medical Association Declaration of Helsinki (World Medical Association, 2013). The School of Psychology ethics committee at the University of Reading (22-064-NW) approved this study prior to recruitment. All participants gave informed consent prior to taking part in the study.

Participants completed the study via Qualtrics, an online survey platform. Analysis of the studies was conducted in R (version 1.4.1106) and SPSS (versions 27, 29).

## Materials

### Videos

We manipulated the framing of the communications shown to participants to reflect one of two motivational styles: autonomy-supportive (i.e., buy-in) or controlling (i.e., pressuring, demanding) communications. Additionally, the videos included one of two types of content focused on motivating behavior: highlighting profits or emphasizing laws and regulations. This resulted in four distinct video-based conditions: (1) Autonomy-supportive framing with profit-focused content, (2) Autonomy-supportive framing with law-focused content, (3) Controlling framing with profit-focused content, and (4) Controlling framing with law-focused content. These communications were presented to participants in the form of videos including audio and images, created by researchers on the project in collaboration with partners in the industry. Each video was under five minutes long.

### Materials check

To conduct materials check, we asked participants whether the video communications viewed were (i) accessible (“clear and easy to understand”), (ii) engaging (“interesting and held my attention”) and (iii) informative (“new and valuable information”) by requesting them to rate their agreement with these three statements communicated using a 7-point Likert scale (-3 = *highly disagree*; 0 = *neutral*; 3 = *highly agree*). We also asked them to indicate whether they have watched the entire video and to summarise the content of the video (in order to exclude those who may have misunderstood the content of the video).

### Manipulation check

For the pilot study, participants answered six statements in a 7-point Likert scale (-3 = *highly disagree*; 0 = *neutral*; 3 = *highly agree*). Example manipulation check question is “How much did you feel the video pressured people to act in certain ways?”. For the main study, a manipulation check was conducted with five questions on a 7-point Likert scale (-3 = *highly disagree*; 0 = *neutral*; 3 = *highly agree*). Example manipulation check question is “The video communication felt supportive”.

### Ethical voice

We measured ‘Ethical Voice’ - the extent to which participants were prepared to act on behalf of transparency value violations (Huang & Paterson, 2017) - by asking participants to rate their agreement with three statements out of six (presented at random) relating to behavioural intentions using a 7-point Likert scale (-3 = *highly disagree*; 0 = *neutral*; 3 = *highly agree*). Example statement is “In the future I would be prepared to talk to a supervisor who wanted to keep bias under wraps / a secret”. The scale had reliability of alpha = .72, 95% CI [.66, .77] in the first study, alpha = .73, 95% CI [.66, .78] in the second study.

### Antagonism

We measured ‘Antagonism’ - the extent to which participants hold a critical perspective regarding the need for transparency - by asking participants to rate their agreement with three statements out of eight (presented at random) relating to how information on bias was communicated using a 7-point Likert scale (-3 = highly disagree; 0 = neutral; 3 = highly agree).

### Motivation

We adapted a measure of internal and external motivations from Legault et al. (2007). Participants rated their agreement with five statements relating to their motivation to transparently communicate bias in their technology using a 7-point Likert scale (-3 = *highly disagree*; 0 = *neutral*; 3 = *highly agree*). Two statements related to internal motivation; two statements related to external motivation. A final statement related to amotivation was not tested here. An example statement of internal motivation is “I value transparency”, whereas an example statement of an external motivation is “I wouldn’t want people to think I’m not transparent”. This measure was only used in Study 2. Correlation between the questions for internal motivation was *r* = .57, *p* <.001, for external motivation *r* = .41, *p* <.001.

### Behavioral intention

The primary outcome variable concerned participants’ behavioral intention in line with transparency values. Participants responded to the question: “If a course were to be offered to me on how to design AI systems in line with transparency and equality values, and on what solutions can be implemented to promote transparent and fair AI systems (such as using auditing meta-toolkits) I would say…” by selecting one of two possible choices: (“Yes, sign me up” – coded as 1 or “No, thank you”-coded as 2). This measure was obtained after reading a short text about what meta-toolkits are. The text was as follows:

“There are toolkits that can be used to evaluate bias in our algorithms. But these are not perfect, either. There is no such thing as a ‘perfectly fair’ algorithm or a toolkit to achieve it. Rather, different ways of measuring bias and fairness are based on different values and can introduce their own problems. These tools can also be used deceptively or to harm rather than help, for example by focusing on certain biases at the expense of others.

Certain metrics, for example, may take pre-existing biases and inequality for granted, and only try not to make things worse going forward. Other metrics will not assume that the status quo is neutral. Some metrics focus solely on accuracy, for example by minimising false positives. Still others care more about how possible outcomes are distributed across groups rather than accuracy or error rates. Each of these types of metrics can be valid depending on the context and ethical or legal requirements.

Ultimately, if we want to understand the full set of limitations in our work and the ways we measure bias and fairness, we use a meta-toolkit. A meta-toolkit can help us evaluate the strengths and weaknesses of different bias tools and metrics, help us choose the right tool for the job, and catch times when tools are being used in a misleading, deceptive, or otherwise unacceptable fashion. In other words, a meta-toolkit helps us use bias and fairness tools as effectively and honestly as possible. It helps us test our existing tools, better understand their limitations, and change how we deploy them individually or in combination to give us the fullest and most accurate possible picture of bias in our systems.”

### Technical knowledge

We asked participants: (i) ‘How do you rate your technical knowledge about AI technology’ and (ii) ‘How familiar are you with the clinical and healthcare applications of AI systems, providing scales from 1 (“*very limited knowledge*”) to 7 (“*expert knowledge*”).

### Demographics and background

We asked participants to describe their gender, ethnicity, age, current educational qualification, and affiliations with healthcare AI. We also asked them about their current plans for possible future careers.

## Pilot Study

### Participants, Procedure, and Analytic Strategy

Using G*Power (Faul et al., 2007), we estimated that the required sample size was a minimum of 112 participants to conduct ANOVA, with an effect size of .04, a power of .95, an alpha level of .05, and four groups. The pilot study involved 121 participants recruited via Prolific with a mean age of 27.15 years, *SD* = 8.71, range = 18 - 60. Eighty-seven participants were male, 31 were female, one was non-binary, and one person identified as ‘other’.

Following reading the study information sheet and providing consent, participants were randomly allocated to watch one of four video communications, which manipulated communication type (autonomy-supportive versus controlling) and motivation type (law versus profit). Following exposure to the videos, participants had to answer a range of questions about video communication designed for a manipulation check, as well as about their background and demographics.

Analysis of the pilot study was conducted in SPSS (version 27). We analyzed pilot study using one-way analysis of variance (ANOVA).

## Results

The pilot study demonstrated that in autonomy-supportive conditions, participants perceived themselves to be more educated about the law (*F* (3,120) = 12.98, *p* < .001). When split into two groups (autonomy-supportive versus controlling communications), exploratory analysis also indicated that autonomy-supportive conditions gave people choice about how to act (i.e., they were effectively supportive, *F* (1,120) = 5.42, *p* = .022) and were more educational about law (F (1,120) = 4.23, *p* = .042). Further, when split into two groups (law versus profit), exploratory analysis indicated that law conditions educated people more about law (*F* (1,120) = 31.26, *p* < .001).

### Study 1

### Participants, Procedure, and Analytic Strategy

Using G*Power (Faul et al., 2007), we estimated that the required sample size was a minimum of 185 participants for a two-tailed linear regression analysis, an effect size of .04, a power of .95, an alpha level of .05, four groups, and three response variables. Participants were recruited via Prolific, with a small number (*N* = 16) being recruited at university lectures. Initially, we recruited 344 participants. However, seven participants were excluded because they did not watch the entire video communication, two participants were excluded from the analysis as they inaccurately summarised the video, 24 were excluded because their degree was not relevant to computer science, and 40 were excluded because they provided blank responses. The final sample of *N* = 271 had *M*age = 24.79 years, *SD* = 5.99, range = 18 - 58. Of these, 145 participants were male, 118 were female, six were non-binary, and two did not wish to disclose their gender.

Following watching the videos and answering manipulation check questions, participants answered questions relating to ethical voice and antagonism (described above). Participants then read a short text about meta-toolkits and asked if they would be willing to take a course to learn more about them (behavioral intention measure). They then completed the demographic questionnaire.

First part of the analysis was conducted in SPSS (version 29). First, we examined correlations between the continuous variables and performed an odds ratios analysis to examine the relationship between behavioral intention and other variables. Then, a one-way ANOVA was conducted to examine whether there are any interactions between autonomy/mandate and profit/law on the manipulation checks. Our primary analysis involved a two-way 2X2 multivariate analysis of variance (MANOVA), investigating the influence of our independent variable (IV) ‘motivational framing’ (with two levels: autonomy-supportive and controlling) and ‘incentive’ (with two levels: economic self-interest and law) on our continuous dependent variables (DVs; post-survey measures: ethical voice and antagonism).

Second part of the analysis was conducted in R (version 1.4.1106). In addition, we conducted a binary logistic regression using the R package glm to examine the effect of communication type conditions (autonomy-supportive versus controlling X economic self-interest versus law) on our binary DV (intention to attend course1 – yes, 2 - no). Model: glm (behavioural intention~ communication type, family = binomial).

### Results

Correlations and odds ratios for study 1 can be found in Table 1. As behavioral intention is a dichotomous variable, for the relationship between behavioral intention and other variables we present odds ratios; for all the other variables in the table, the presented numbers represent correlation coefficients.

Manipulation check did not reveal significant differences between the conditions. There was no effect of condition on antagonism (*F*(3, 259) = 0.37, *p* = .773), ethical voice (F(3, 259) = 0.16, *p* = .922), or behavioral intention (*b* = .04, *SE* = .03, *p* = .092).

### Brief Discussion

Study 1 did not show significant difference between communication type (autonomy-supportive or controlling) or communication framing (emphasizing legal implications versus emphasizing profit) in predicting participants’ behavioral intention to engage in anti-bias training. However, importantly, the manipulation check indicated that manipulation was not successful. Therefore, we introduced strategic changes to wording of questions in the following study to amplify the effects of communication type on perceptions of motivational framing.

Furthermore, we found that both ethical voice and familiarity with healthcare AI were positively correlated with behavioral intention, suggesting that participants who were more willing to speak up about transparency violations and those who has more experience with healthcare AI were also more likely to express an intention to participate in anti-bias training. These results indicated that participants who care about transparency are more likely to engage in an educational course. However, we did not directly measure participants’ motivations to act in line with the value of transparency. Therefore, Study 2 aimed to expand the results of Study 1 by examining participants’ motivation type (i.e., internal or external).

### Study 2

The manipulation in Study 1 was not successful, and the results did not indicate that communication type was associated with the measured outcomes. To address this, we made several changes to the methodology for Study 2 to improve the manipulation and better assess the impact of the videos. The wording of the questions and the text around the videos was adjusted to make the communications either more controlling or more supportive, and to further emphasize points about law and profit. For example, in controlling conditions, participants were asked to consider “why it is morally flawed and shameful” to not be transparent, while in supportive conditions they were prompted to think “how the content is personally meaningful to you.” Similarly, in law conditions, participants were asked to consider “why it should be mandated to be transparent.” Participants were also required to summarize the videos after watching them to ensure they understood the content, and those who did not were excluded. Additionally, two key modifications were made to the procedure in Study 2. First, a fifth “comparison” group was introduced, in which participants did not watch a video but answered the remaining questions. This addition allowed us to better isolate the impact of video content on participants’ attitudes and perceptions while serving as a control group to enhance the robustness of our comparative analysis. Second, a motivation measure was added to assess how internal or external motivation could be associated with behavioral intention. Internal and external motivations were included as potential predictors of behavioral intention based on Self-Determination Theory to help unpack the underlying drivers of participants’ decisions. Here, internal motivation reflects a genuine valuing of transparency, while external motivation captures the influence of external pressures or expectations (Ryan & Deci, 2020). By measuring these constructs, we sought to determine whether internal or external factors, or both, were associated with participants’ willingness to engage with the proposed meta-toolkit course. This addition allowed us to expand on the findings of Study 1 and explore the broader psychological landscape shaping behavioral intentions.

### Participants, Procedure, and Analytic Strategy

Using G*Power (Faul et al., 2007), we estimated that the required sample size was a minimum of 185 participants for a two-tailed linear regression analysis, an effect size of .04, a power of .95, an alpha level of .05, five groups, and four response variables. We initially recruited 245 participants via Prolific. However, two participants were excluded because they did not watch the entire video, two were excluded because they failed the attention check question, and 30 were excluded because they provided blank responses, leaving a sample of 209 participants. Mean age of participants was 24.74, *SD* = 5.62, range = 18 - 55. 103 participants were male, 95 were female, nine were non-binary, and two did not wish to disclose their gender.

Analysis of Study 2 replicated analytic strategy of study 1. We also ran an exploratory analysis investigating how communication type was associated with motivation (by performing MANOVA in SPSS, version 29). Further, we use R (version 1.4.1106) to perform an exploratory analysis examined how motivation was associated with behavioural intention (R code being [glm (behavioural intention~ internalmotivation, family = binomial)]; [glm (behavioural intention~ externalmotivation, family = binomial)]). Exploratory analysis compared these models by using ‘coef’ function that extracted the coefficients and standard errors and then calculated the test statistic and *p*-value for the comparison. In a similar way, exploratory analysis examined how autonomy and control motivations were associated with ethical voice and antagonism.

## Results

### Correlations and odds ratios for Study 2 can be found in Table 2

One-way ANOVA revealed significant differences between the conditions based on whether the video made participants interested in the topic it was focused on (F (3, 159) = 2.84, *p* = .040). Pairwise comparisons indicated that supportive-law communications were rated as significantly more interesting than controlling-law communications (*p* = .025).

There was no effect of condition on antagonism (*F* (4, 203) = 2.21, *p* = .069), ethical voice (*F* (4, 203) = .553, *p* = .697), or behavioural intention (*b* = -.003, SE = .022, *p* = .877).

However, exploratory analysis demonstrated that both internal (*b* = -.05, SE = .02, *p* = .002) and external (*b* = -.034, SE = .013, *p* = .011) motivations were associated with behavioral intention to take part in a meta-toolkit course.^1^ The difference between the beta coefficients was -0.02, SE = .02, *p* = .423, indicating that internal and external motivations are not significantly different from each other in predicting behavioral intention. In addition to these findings, greater internal (*b* = .19, SE = .039, *p* <.001) and external (*b* = .11, SE = .03, *p* <.001) motivations were associated with greater ethical voice and with lower antagonism (*b* = -.13, SE = .05, *p* = .013 for internal; *b* = -.09, SE = .04, *p* = .043 for external). Exploratory analysis indicated that communication type was not a significant predictor of motivation type.

### Brief Discussion

Similarly to Study 1, communication type was not a predictor of behavioral intention. Further, communication type did not significantly affect participants’ levels of antagonism or ethical voice. This suggests that other factors, such as individual differences (Lofaro et al., 2023) or societal pressures (van Nuspeet et al., 2025), may play a greater role in shaping participants’ commitment to anti-bias behaviors.

However, In Study 2 we found that both internal motivation and external motivation were associated with participants’ behavioral intention to take part in an anti-bias course. These findings indicate that both internal and external motivations play a role in shaping individuals’ intentions to engage in anti-bias training. They may indeed have additive effects such that the more motivation, regardless of its type, the more behavioral intention within this context.

Similarly, greater internal and external motivations for transparency were associated with higher levels of ethical voice - willingness to advocate for transparency - and lower levels of antagonism - critical perspective regarding transparency. In all, motivating students, and potentially developers themselves, to adopt anti-bias training could influence their behavioral intention to take part in value-driven training and promote ethical behaviors, such as speaking out against bias (Dwyer & Faber-Langendoen, 2018).

### General discussion

The current well-powered experiments investigated how brief communications, framed either in supportive or mandating manner, and either in terms of reward (personal gain) or law (a negative incentive), are associated with technology students’ willingness to take a course on transparent communication about potential biases in healthcare AI technology. We hypothesized that the autonomy-supportive motivational framing conditions would predict more positive behavioral intention when compared to those conditions in which motivation was conveyed in a controlling way. We also hypothesised that those in profit condition (i.e., where self-interest is highlighted) would exhibit more positive behavioral intention than those in law condition (i.e., where potential losses are highlighted). Further, we predicted that buy-in would magnify beneficial effects of law on positive behavioral intention and beneficial effects of self-interest on positive behavioral intention. The hypotheses were not supported, as we did not find conditions to have an effect on behavioral intentions.

The findings of this study suggest that neither controlling nor supportive communication are associated with the willingness of technology students to engage in education courses regarding transparent communication about bias in technology. They imply that the brief communication approaches, regardless of whether they are controlling or supportive in nature, may not be effective in fostering the desired level of engagement in educational initiatives focused on addressing bias in technology. Similar weak and null effects were found in a recent large-scale study of individuals receiving public health messages concerned with the Covid-19 Pandemic (Legate et al., 2022) and Type 2 diabetes (Farmer et al., 2016), as well as in examining brief autonomy-supportive instructions for the enjoyment of solitude (Nguyen et al., 2022). Therefore, the evidence that brief communication framing has a potential to impact people’s motivation is lacking, and future research should investigate longer forms of communication as a driver for behavior.

Further, although there is not much literature to speak to this, the findings reveal that neither positive (gaining profit) nor negative (being punished by law) consequences were associated with students’ willingness to engage in a course about transparent communication, or with motivation type. Thus, alternative, and potentially more long-term strategies need to be explored to enhance the motivation and willingness of technology students to actively participate in educational projects aimed at promoting transparent communication and addressing bias within the field, ultimately benefiting the end-user.

While communication type was not associated with motivation type, our study did find that both higher internal and external motivation were associated with willingness to take a course on how to communicate bias in technology. These findings suggest that individuals who find it personally important to *them* to pursue transparency (internal motivation) and those who are driven by external factors such as rewards, recognition, or career aspirations (external motivation) are more likely to demonstrate a proactive attitude toward addressing bias in technology. Our exploratory analysis indicated that there was no significant difference between the extent to which internal and external motivation predict behavioral intention. This contributes to the existing body of literature regarding the efficiency of external and controlled motivation in motivating behavior, the results of which have to date been inconclusive (e.g., Benita et al., 2023; Legate et al., 2019).

However, the current study indicates that increasing motivation may not be achievable through brief communication interventions. Instead, it might be a longer-term process that requires sustained efforts and continuous reinforcement. Thus, exploring the potential benefits of continuous communications within the workplace or educational settings may prove fruitful in enhancing individuals’ motivation to address bias in technology. Indeed, previous research found that consistent autonomy-supportive, as opposed to controlling, leadership is associated with increased employee’s engagement (Sarmah et al., 2022; Slemp et al., 2018). Regular discussions, workshops, or mentoring programs could help organizations to create an environment that nurtures and sustains individuals’ motivation, thereby promoting a deeper understanding of bias and developing transparent communication practices.

In both Study 1 and Study 2, we found that behavioral intention was positively correlated with ethical voice and technical familiarity, suggesting that individuals who are more willing to speak up about ethical issues and who have greater familiarity with AI systems are more likely to take actionable steps toward adopting transparency practices. Further, in Study 2, the positive association between ethical voice and both internal and external motivations suggests that individuals who are willing to act against transparency violations are also more motivated, both intrinsically and extrinsically, to promote transparency. These findings align with previous research indicating that ethical voice reflects a proactive stance toward addressing organizational issues and advocating for change (Huang & Paterson, 2017). They specifically reinforce the idea that ethical commitment may serve as a driving force behind the willingness to engage in pro-social behaviors. In contrast, the negative relationship between antagonism and motivation indicates that negative attitudes may reduce individuals’ readiness to act congruently with their transparency values. Prior work suggests that antagonistic attitudes can undermine foster disengagement and resistance and decrease behavioral intention (Hauptman et al., 2024). Together, these findings suggest that raising both ethical awareness and technical competence could strengthen motivations and intentions to engage with tools and practices that promote fairness and transparency. The results of this study support the idea that organizational climate that aims to cultivate internal and external motivation of the employees is a pivotal factor in shaping the values of fairness and inclusion and associated behaviors and employee’s performance (Italiani et al., 2022; Syarief et al., 2022). A consistently positive organizational climate, characterized by the values of trust, transparency, and respect, cultivates internal motivation of employees to embrace and uphold these values (Men & Stacks, 2014). Providing feedback, recognizing, and rewarding behaviors that are consistent with organisational values, having formal ‘code of conduct’, as well as setting informal norms, leading by example, and being aware regarding individual differences among employees help leaders of the organisations help to cultivate desired attitudes and behaviors (Besio & Pronzini, 2014; Grojean et al., 2004). Such a climate, referred to in the literature as ‘ethical leadership’, not only reinforces ethical conduct and adherence to core values but also fosters a sense of belonging and commitment among employees (Avey et al., 2012). In this way, a conducive organizational climate becomes a cornerstone for nurturing the values of fairness, inclusion, and transparency and behaviors that drive an organization toward its goals and mission, ultimately contributing to its success and sustainability and, by extension, better healthcare.

Indeed, the characteristics of good-quality healthcare, as well as diagnostic and treatment technology, include fairness and inclusion. By adhering to these values, organisations developing healthcare AI can contribute to reducing healthcare disparities, improving access to quality care, reducing costs, and enhancing patient outcomes (Abràmoff et al., 2021; Al-Mufti et al., 2019; Wahl et al., 2018). Transparency about the fairness and inclusion in healthcare AI systems can foster trust among healthcare professionals and patients alike, reinforcing the idea that AI is a valuable tool in augmenting healthcare delivery (Richardson et al., 2021; Shinners et al., 2020).

### Limitations and Future Directions

Several limitations should be considered when interpreting the findings. First, we used technology students, rather than technology developers, as our sample. While this choice was made to minimize prior knowledge and opinions on the subject, as students, the presented consequences may not carry the same weight or real-world implications as they would for experienced professionals. Consequently, the generalizability of the findings to the actual technology development industry may be limited.

Another limitation is that there were no significant differences in conditions when manipulation check was conducted. This suggests that the brief communication interventions employed in this study may not have effectively influenced participants’ motivations as intended. It is unclear whether this is due to the efficacy of the interventions themselves or whether motivation simply could not be shifted through a brief communication. Thus, future research should focus on refining such communication interventions to enhance their impact and increase the likelihood of detecting significant effects.

Finally, it is important to acknowledge the low correlation between the items within internal and external motivations subscales. This score reflects the consistency and stability of the measurements, and a low score suggests potential measurement limitations.

## Conclusion

Our study sheds light on the complex interplay between communication type, motivation type, and willingness to engage in education about transparent communication regarding bias in technology. While we did not find a direct association between communication type and motivation, the links between higher intrinsic and extrinsic motivation, and willingness to participate in bias communication education and speak up for transparency values, highlights the importance of motivation as a catalyst for transparency.

## Figures and Tables

**Table 1:** Correlations and Odds Ratios Between the Main Variables in Study 1

Variable	Behavioral Intention	Ethical Voice	Antagonism	Technical Knowledge	Familiarity with Healthcare AI
**Behavioral Intention**	—	0.06, 95% CI [0.45, 0.76] ***	1.08, 95% CI [0.88, 1.33]	1.04, 95% CI [0.81, 1.35]	0.69, 95% CI [0.53, 0.90] ***
**Ethical Voice**		—	-0.02	0.02	0.11
**Antagonism**			—	0.08	-0.02
**Technical Knowledge**				—	0.51 ***
**Familiarity with** **Healthcare AI**					

* *p* < .05, ** *p* < .01, *** *p* < .001

*Note:* For the relationship between behavior intention and other variables we present odds ratios; for all the other variables in the table, the presented numbers represent correlation coefficients. Higher scores represent lower behavioral intention.

**Table 2:** Correlations and Odds Ratios Between the Main Variables in Study 2

Variable	Behavioral Intention	Ethical Voice	Antagonism	Technical Knowledge	Familiarity with Healthcare AI	Internal Motivation	External Motivation
**Behavioral Intention**	—	0.78, 95% CI [0.57, 1.06]	0.91, 95% CI [0.43, 0.72]	1.06, 95% CI [0.80, 1.47]	0.96, 95% CI [0.71, 1.28]	0.82, 95% CI [0.67, 1.01]	0.94, 95% CI [0.80, 1.12]
**Ethical Voice**		—	-0.12	0.09	0.07	0.32 ***	0.23 ***
**Antagonism**			—	0.06	0.13	-0.17 *	-0.14 *
**Technical Knowledge**				—	0.61 ***	0.15 *	0.04
**Familiarity with** **Healthcare AI**					—	0.08	-0.00
**Internal Motivation**						—	0.54 ***
**External Motivation**							—

* *p* < .05, ** *p* < .01, *** *p* < .001

*Note:* For the relationship between behavior intention and other variables we present odds ratios; for all the other variables in the table, the presented numbers represent correlation coefficients. Higher scores represent lower behavioral intention.

## Data Availability

Materials, data, and analysis code that are associated with this submission could be found on the Open Science Framework: https://osf.io/5uq27/. This study’s design, hypotheses, and analytic strategy were preregistered: https://osf.io/g8y39.

## Footnotes

1 Higher scores indicate lower behavioral intention, as intention to attend the course was coded as 1 – yes, 2 – no.
